# Cross-packaging of genetically distinct mouse and primate retroviral RNAs

**DOI:** 10.1186/1742-4690-6-66

**Published:** 2009-07-14

**Authors:** Noura Salem Al Dhaheri, Pretty Susan Phillip, Akela Ghazawi, Jahabar Ali, Elizabeth Beebi, Soumeya Ali Jaballah, Tahir A Rizvi

**Affiliations:** 1 Department of Microbiology & Immunology, Faculty of Medicine and Health Sciences (FMHS), United Arab Emirates University (UAEU), Al Ain, UAE

## Abstract

**Background:**

The mouse mammary tumor virus (MMTV) is unique from other retroviruses in having multiple viral promoters, which can be regulated by hormones in a tissue specific manner. This unique property has lead to increased interest in studying MMTV replication with the hope of developing MMTV based vectors for human gene therapy. However, it has recently been reported that related as well as unrelated retroviruses can cross-package each other's genome raising safety concerns towards the use of candidate retroviral vectors for human gene therapy. Therefore, using a *trans *complementation assay, we looked at the ability of MMTV RNA to be cross-packaged and propagated by an unrelated primate Mason-Pfizer monkey virus (MPMV) that has intracellular assembly process similar to that of MMTV.

**Results:**

Our results revealed that MMTV and MPMV RNAs could be cross-packaged by the heterologous virus particles reciprocally suggesting that pseudotyping between two genetically distinct retroviruses can take place at the RNA level. However, the cross-packaged RNAs could not be propagated further indicating a block at post-packaging events in the retroviral life cycle. To further confirm that the specificity of cross-packaging was conferred by the packaging sequences (ψ), we cloned the packaging sequences of these viruses on expression plasmids that generated non-viral RNAs. Test of these non-viral RNAs confirmed that the reciprocal cross-packaging was primarily due to the recognition of ψ by the heterologous virus proteins.

**Conclusion:**

The results presented in this study strongly argue that MPMV and MMTV are promiscuous in their ability to cross-package each other's genome suggesting potential RNA-protein interactions among divergent retroviral RNAs proposing that these interactions are more complicated than originally thought. Furthermore, these observations raise the possibility that MMTV and MPMV genomes could also co-package providing substrates for exchanging genetic information.

## Background

The mouse mammary tumor virus (MMTV) is a betaretrovirus that has been primarily implicated in causing breast cancer and, to some extent, T-cell lymphomas in mice, reviewed in Mustafa et al., [1,2]. Classically, MMTV has been categorized as a simple retrovirus containing only the structural and regulatory genes needed to complete the virus life cycle. However, recently, it has been proposed that MMTV be reclassified as a more complex murine retrovirus because of the presence of accessory/regulatory factors such as *sag *[3,4], *Naf *[5], and the recently identified Rem/RmRE regulatory pathway of MMTV [6-8]. In addition, it has recently been shown that Rev and Rex proteins of human complex retroviruses can interact with MMTV Rem responsive element [9]. Furthermore, MMTV is unique from other simple and complex retroviruses in harboring several promoters for the expression of its various gene products [10-12]. Of these several promoters, two have been identified in the long terminal repeats (LTR) and two in the envelope region, reviewed in Mustafa et al., [1]. The LTR promoters are under the influence of steroid hormones in a tissue-specific manner due to the presence of hormone responsive elements (HREs), making them inducible [13]. Therefore, the tissue-specific and inducible MMTV promoters have lead to increased interest in studying MMTV replication with the ultimate goal of developing MMTV based vectors for their potential use in targeted gene therapy. In one recent study, MMTV promoters have been utilized in murine leukemia virus (MLV) based vectors used for targeted enzyme prodrug therapy both *in vivo *and *in vitro *[14]. The use of MMTV based vectors would not only provide tissue-specific and inducible expression of the therapeutic gene, but also its non-primate nature may circumvent potential safety concerns. Such concerns include cross-packaging and co-packaging of the transfer vector RNA genome by related primate retroviruses or retrovirus-like elements resulting in the generation of recombinant variants with unknown pathogenic potential.

Packaging of retroviral genomic RNA by the assembling virus particles is a crucial step in the virus life cycle. RNA packaging among retroviruses is unique and a highly specific phenomenon during which two copies of full length "unspliced" genomic RNA are preferentially packaged from amongst a wide pool of cellular and other spliced viral RNAs, reviewed by D'Souza and Summers [15] and Lever [16]. The specificity towards RNA packaging by the newly assembling viral particles is conferred by the recognition of specific *cis*-acting sequences, the packaging signal (ψ), present at the 5'end of the viral genome, and the nucleocapsid (NC) protein is responsible for discriminating between spliced and unspliced viral RNA, reviewed by D'Souza and Summers [15] and Lever [16]. Despite this specificity, in some cases, it has been shown that evolutionary related, however molecularly different, retroviruses can cross- and co-package each other's genome suggesting that phylogenetically related retroviruses are capable of using similar protein-and RNA-packaging elements, reviewed by D'Souza and Summers [15] and Lever [16]. Such cross- and co-packaging among retroviruses have been shown to exchange genetic information resulting in recombinant variants [17,18] undermining many advantages of using retroviral vectors in human gene therapy studies. The use of phylogenetically distant non-human retroviral vectors, such as those based on MMTV, should minimize the chances of recombination with unrelated primate exo-and/or endogenous retroviruses.

In spite of the advantages of using non-human retroviral vectors with inducible tissue-specific promoters as in the case of MMTV, so far, no detailed studies have been conducted to investigate the ability of MMTV RNA to be cross-packaged in human cells by heterologous primate retroviral proteins. However, in one earlier report, Günzburg and Salmons have reported that MMTV RNA could not be packaged by Moloney murine leukemia virus (MoMLV) packaging cell lines [19]. Such limited information regarding MMTV RNA packaging and cross-packaging studies in the literature can be attributed to 1) a *trans *complementation assay for MMTV was not developed until recently, and 2) MMTV is not expressed very efficiently in human cells due to the promoter's low transcriptional activity. In order to overcome these drawbacks, we have successfully replaced the U3 region of MMTV 5'LTR with the human cytomegalovirus (hCMV) promoter in MMTV based vectors to allow for its efficient expression in human cells; and we have also developed a three-plasmid *trans *complementation assay for MMTV to study its RNA packaging and propagation [20]. Using this *in vivo *packaging and transduction assay, we investigated the ability of MMTV RNA to be cross-packaged by a primate retrovirus, the Mason-Pfizer monkey virus (MPMV) that, like MMTV, also preassembles in the cytoplasm before budding. Our results showed that both of these viruses could cross-package each other's RNAs. However, the cross-packaged RNA could not be propagated further and therefore failed to transduce the target cells suggesting a block at post RNA packaging events of the retroviral life cycle such as reverse transcription and/or integration. Our results further demonstrated that this cross-packaging specificity was conferred specifically by the packaging sequences, which were in turn recognized by the heterologous proteins; since cloning of these sequences in plasmids, which generate non-viral RNAs, resulted in the encapsidation of these non-viral RNAs by MMTV and MPMV proteins reciprocally. The results presented in this study strongly suggest that MPMV and MMTV are promiscuous in their ability to cross-package each other's genome and that interactions between retroviral RNAs and Gag polyprecursors are more complicated than originally thought.

## Results and discussion

### *In vivo *packaging and transduction assay for MMTV and MPMV

**Figure 1:**
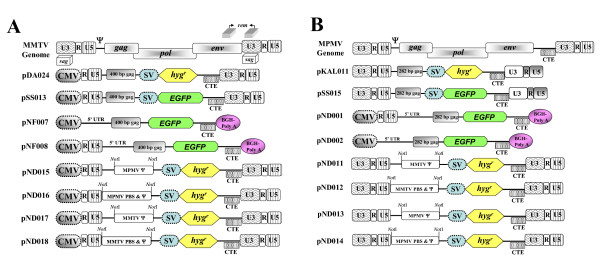
**Schematic representation of MMTV and MPMV based vectors**. **(A) **MMTV genome, MMTV transfer vectors, and expression plasmids containing MMTV or MPMV packaging signal. **(B) **MPMV genome, MPMV transfer vectors, and expression plasmids containing MPMV or MMTV packaging signal. The design and construction of these vectors is described in materials and methods and can be further obtained from authors upon request. CMV, human cytomegalovirus promoter; SV, Simian virus 40 promoter; *hyg*^*r*^, *hygromycin resistance *gene; CTE, constitutive transport element from Mason-Pfizer monkey virus (MPMV); *EGFP, enhanced green fluorescence protein *gene; BGH, bovine growth hormone.

To study cross-packaging between MMTV and MPMV, we used three-plasmid *trans *complementation assays developed earlier by our laboratory [20,21]. These MMTV and MPMV *trans *complementation assays consist of a packaging construct, pJA10 or pTR301, which expresses either MMTV or MPMV *gag/pol *genes, respectively, which results in the production of viral particles, which are capable of encapsidating viral RNA containing ψ. The source of the packageable RNA is provided by MMTV (pDA024, pSS013) and MPMV (pKAL11, pSS015) transfer vectors (Figure 1A and 1B). These transfer vectors contain the sequences responsible for RNA packaging, in addition to the *cis*-acting sequences needed for viral replication, which include Primer Binding Site (PBS), Poly Purine Tract (PPT) (needed for reverse transcription), and U3 and U5 attachment (*att*) sites (required for integration). In addition, these transfer vectors express *hygromycin resistance *and/or *enhanced green fluorescence protein *(*EGFP*) gene from an internal simian virus (SV40) promoter (SV-*hyg*^*r*^/SV-*EGFP*), which allows for the monitoring of the successful propagation of the transfer vector RNAs by transducing the target cells with these marker genes. An envelope expression plasmid (MD.G) based on vesicular stomatitis virus envelope G (VSV-G) was also used to enable the study of steps involved in both packaging and propagation of the transfer vector RNAs [22].

Briefly, in these assays, the three plasmids are co-transfected into 293T producer cells, which will generate virus particles containing the encapsidated RNA, the replication of which is limited to a single round because reinfection of the target cells cannot take place. These virus particles can be used to: 1) directly examine the viral RNA content in the virus particles using reverse transcriptase polymerase chain reaction (RT-PCR) and 2) to infect target cells resulting in the transduction of these cells with the marker gene, thus allowing for monitoring the propagation of the transfer vector RNA. The number of Hygromycin-resistant (Hyg^r^) colonies and/or EGFP positive cells obtained should be directly proportional to the amount of RNA that is packaged into the virus particles providing an indirect estimate of RNA packaging.

### MMTV RNA can be cross-packaged but cannot be propagated by MPMV proteins

To determine whether MMTV RNA can be cross-packaged by the heterologous primate retrovirus (MPMV) proteins, we co-transfected MMTV transfer vectors (pDA024 and pSS013) with MPMV packaging construct (pTR301) along with the envelope expression plasmid (MD.G) into 293T cells. As a control, MMTV transfer vectors were also co-transfected with the homologous packaging construct (pJA10) along with the envelope expression plasmid. Supernatants from the transfected cultures were used to isolate the viral RNA to determine vector RNA packaging and to infect human HeLa CD4+ cells in order to study vector RNA propagation.

**Figure 2:**
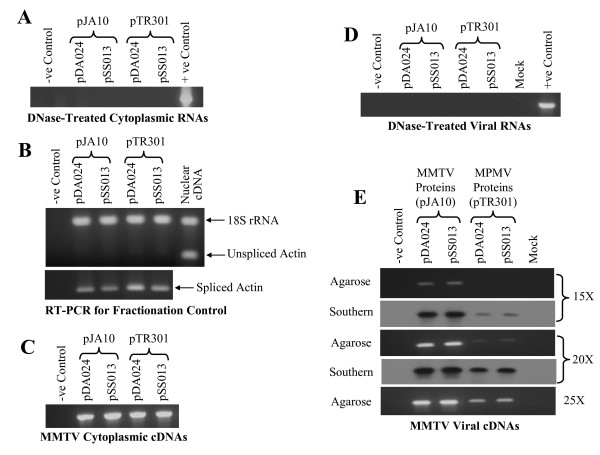
**MMTV transfer vectors RNA can be cross-packaged by MPMV proteins**. **(A) **PCR amplification of cytoplasmic RNAs treated with DNase to ensure the absence of any contaminating DNA in the RNA preparations using primers OTR537 and OTR538. **(B) **Control for nucleocytoplasmic RNA fractionation technique to ensure that the transfer vector RNAs were properly transported to the cytoplasm. Upper panel represents the multiplex RT-PCR for unspliced β-actin mRNA (found exclusively in the nucleus) and 18S ribosomal RNA as a control for the presence of amplifiable cDNA in the PCR reactions as described in the materials and methods and results sections. Unspliced β-actin was not detected in the cytoplasmic RNA fraction, ensuring that the transfer vector RNAs were properly transported to the cytoplasm without any compromise on the integrity of the nuclear membrane. The lower panel represents RT-PCR on cytoplasmic RNA for spliced β-actin mRNA and should be present in both nuclear and cytoplasmic fractions. **(C) **RT-PCR of cytoplasmic cDNA amplified using MMTV specific primers to ensure that the transfer vector RNAs were stably expressed. **(D) **PCR amplification of DNase treated viral RNAs to confirm the absence of any contaminating plasmid DNA carried over from the transfected cultures. **(E) **RT-PCR of viral cDNAs amplified using virus specific primers and probed with the PCR product amplified using the same set of primers and HYB MTV as a template. Amplifications were conducted for 15, 20, and 25 cycles and, in addition to agarose gel, Southern blots are also shown. For this set of experiment, while amplifying DNase treated viral RNAs, cytoplasmic and viral cDNAs, primers OTR643 and OTR676 were used and should amplify 585 bp fragment.

To ensure that the transfer vector RNAs are efficiently and stably expressed and properly transported from the nucleus to the cytoplasm, RNAs from the transfected cells were fractionated into cytoplasmic and nuclear fractions. To verify that there was no contaminating plasmid DNA in our cytoplasmic RNA preparations, which may confound the interpretation of our results, cytoplasmic RNAs were treated with RNase free DNase and were PCR amplified. The lack of a positive PCR signal, following 30 cycles of amplification, indicated that the contamination in our cytoplasmic RNA preparations is below the detection level (Figure 2A). After making the cDNA, we confirmed that the transfer vector RNAs were properly transported from the nucleus to the cytoplasm by ensuring that no compromise was made on the integrity of the nuclear membrane during the fractionation process based on the absence of unspliced β-actin mRNA in the cytoplasmic RNA fraction as detected by RT-PCR. Unspliced β-actin mRNA is found exclusively in the nucleus, while the spliced form is found in both the nucleus and the cytoplasm [23]. To ensure that each cytoplasmic sample in the unspliced β-actin PCRs contained amplifiable cDNA, therefore, as an ancillary control, PCRs were conducted for 25 cycles in the presence of primers/competimers for 18S ribosomal RNA. Figure 2B shows that there was a total lack of unspliced β-actin message in the cytoplasmic fraction (upper panel) suggesting that there was no leakage of RNA from the nucleus. The presence of spliced β-actin mRNA observed in the cytoplasmic fraction (lower panel) confirmed that the transfer vector RNAs were properly transported to the cytoplasm. To exclude the possibility of poor expression and/or instability of the transfer vector RNAs, cDNAs prepared from the cytoplasmic fractions were amplified using viral specific primers and were found to be stably expressed (Figure 2C).

Having confirmed that all transfer vector RNAs were stably expressed and efficiently transported to the cytoplasm, we examined the ability of MPMV proteins to cross-package MMTV transfer vector RNAs. Like cytoplasmic RNA fractions, viral RNAs were treated with DNase, reverse transcribed, and amplified using viral specific primers for varying number of cycles. In addition, Southern blotting was performed on the PCR products using transfer vector RNA specific probe as described previously [24]. Test of the transfer vector RNAs packaged into the viral particles by RT-PCR revealed that MPMV proteins (pTR301) were able to cross-package MMTV transfer vector (pDA024 and pSS013) RNAs. The cross-packaged RNAs, following RT-PCR, could be visualized by ethidium bromide staining within 20 cycles of PCR, but Southern blotting was needed to appreciate cross-packaging after 15 PCR cycles (Figure 2E). However, the level of cross-packaging efficiency was lower when compared to MMTV vectors (pDA024 and pSS013) packaged by the homologous MMTV proteins (pJA10) (Figure 2E). Taken together, these results demonstrated that MMTV transfer vector RNAs could be cross-packaged by MPMV proteins within the detectable range.

Since our vectors contained a *hygromycin resistance *marker or an *EGFP *gene, we examined the propagation of these vectors by infecting the target cells with supernatants produced by the transfected cells. Following infection, if the vectors were properly propagated, these marker genes (*hygromycin resistance *gene in the case of pDA024 and *EGFP *gene in the case of pSS013) will transduce the target cells resulting in Hyg^r ^colonies or EGFP positive cells suggesting the successful completion of crucial steps of the viral life cycle such as reverse transcription and integration following RNA packaging. As expected, the propagation of the packaged MMTV transfer vector RNAs by homologous MMTV proteins was readily observed as evidenced by the presence of Hyg^r ^colonies in the case of pDA024 and EFGP positive cells in the case of pSS013 (Table 1) indicating that the MMTV transfer vectors are capable of efficiently expressing the marker genes. On the other hand, the lack of Hyg^r ^colonies or EGFP positive cells when MMTV vector (pDA024 and pSS013) RNAs were cross-packaged by MPMV proteins (pTR301) suggested that the cross-packaged RNAs could not be propagated (Table 1).

**Table 1:** Propagation of MMTV and MPMV transfer vectors RNA by homologous and heterologous proteins.

		**Titers (CFU/ml)^a, b^**		**% EGFP** **Positive Cells^c^**	
		
**Transfer Vector**	**Description of the Transfer Vector**	**MMTV Protein (JA10)**	**MPMV Protein (TR301)**	**MMTV Protein (JA10)**	**MPMV Protein (TR301)**
pDA024	Chimeric LTR, 5' region upto 400 bp of MMTV Gag, and SV-*hyg*^*r*^	3,676 ± 196	< 1	-	-

pSS013	Chimeric LTR, 5' region upto 400 bp of MMTV Gag, and SV-*EGFP*	-	-	19	< 1

pND015	Same as DA024 but the putative MMTV ψ has been replaced with that of MPMV (5' UTR + 282 bp of Gag)	2	8	-	-

pND016	Same as DA024 but the putative MMTV ψ and PBS have been replaced with that of MPMV	< 1	7	-	-

pND017	Control MMTV vector in which putative MMTV ψ has been cloned back after creating *Not*I site at PBS/UTR junction	187 ± 33	ND	-	-

pND018	Control MMTV vector in which putative MMTV ψ and PBS have been cloned back after creating *Not*I site at U5/PBS junction	30 ± 4	ND	-	-

pKAL011	MPMV 5' region upto 282 bp Gag and SV-*hyg*^*r*^	< 1	36,373 ± 3,972	-	-

pSS015	Same as KAL011 but has SV-*EGFP *instead of SV-*hyg*^*r*^	-	-	< 1	41

pND011	Same as KAL011 but MPMV ψ has been replaced with that of MMTV (5' UTR + 400 bp of Gag)	< 1	3	-	-

pND012	Same as KAL011 but MPMV ψ and PBS have been replaced with that of MMTV	< 1	23	-	-

pND013	Control MPMV vector in which MPMV ψ has been cloned back after creating *Not*I site at PBS/UTR junction	ND	2813 ± 99	-	-

pND014	Control MPMV vector in which MPMV ψ and PBS have been cloned back after creating *Not*I site at U5/PBS junction	ND	1660 ± 111	-	-

pTR174	Control vector containing SV-*hyg*^*r *^cassette and SIV 3'LTR as poly(A)	< 1	< 1	-	-

pAG001	Control vector containing SV-*hyg*^*r *^cassette and FIV 3'LTR as poly(A)	< 1	< 1	-	-

Mock	No DNA (control)	<1	< 1	< 1	< 1

^a ^CFU/ml, colony forming units per milliliter of non-concentrated supernatant from the transfected cultures. ^b ^Each value represents a mean of three experiments performed in duplicates. ^c ^Percentage of EGFP positive cells represent the phenotypic analysis of the transduced target cells. Single cell suspensions of the infected HeLa CD4+ cells were prepared, and 10,000 events (cells) per group were counted and analyzed using Becton Dickinson FACS. < 1 indicates < 1 Hyg^r ^colonies in the target cells or < 1 EGFP positive cells out of 10,000 events (cells) analyzed.

### MPMV RNA can be cross-packaged but cannot be propagated by MMTV proteins

To determine whether MMTV proteins can cross-package MPMV RNA or not, MPMV transfer vectors (pKAL011 and pSS015) were co-transfected along with MMTV packaging construct (pJA10) and the envelope expression plasmid MD.G. In parallel, as a control, MPMV transfer vectors were also co-transfected with their homologous packaging construct (pTR301) and the envelope expression plasmid.

**Figure 3:**
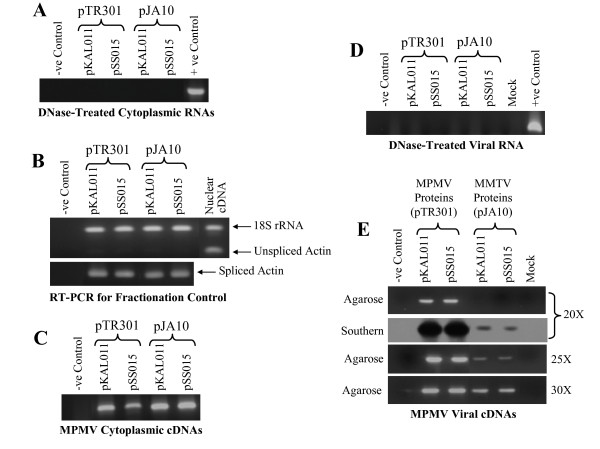
**MPMV transfer vectors RNA can be cross-packaged by MMTV proteins**. **(A) **PCR amplification of DNase treated cytoplasmic RNAs using primers OTR537 and OTR538. **(B) **Control for nucleocytoplasmic RNA fractionation technique as described for figure 2B. **(C) **RT-PCR of cytoplasmic cDNA amplified using MPMV specific primers confirming that the transfer vector RNAs were stably expressed. **(D) **PCR amplification of DNase treated viral RNAs using viral specific primers. **(E) **RT-PCR of viral cDNAs amplified (for 20, 25, and 30 cycles) using virus specific primers and probed with the PCR product amplified using the same set of primers and pKAL011 as template. For this set of experiments, while amplifying DNase treated viral RNAs, cytoplasmic and viral cDNAs, primers OTR216 and OTR263 were used and should amplify 271 bp fragment.

After confirming the absence of any contaminating plasmid DNA in our cytoplasmic and viral RNA preparations (Figure 3A and 3D), we confirmed that all MPMV transfer vector RNAs were efficiently transported to the cytoplasm and were stably expressed (Figure 3B and 3C). Next, we investigated the ability of MPMV RNA to be cross-packaged by MMTV proteins by directly examining the viral RNA content in MMTV particles. RT-PCR results in figure 3E demonstrated that MPMV transfer vector (pKAL011 and pSS015) RNAs were cross-packaged by MMTV proteins (pJA10). Consistent with the results obtained for MMTV RNA cross-packaging, the efficiency of the cross-packaged MPMV vector RNAs was lower when compared to the homologous MPMV vector (pKAL011 and pSS015) RNAs being packaged by its own proteins (pTR301) (Figure 3E). The cross-packaging efficiency of MPMV RNA by MMTV proteins appeared to be lower when compared to MMTV RNAs being cross-packaged by MPMV proteins since Southern blotting was needed to appreciate cross-packaging after 20 cycles of PCR instead of the 15 cycles needed to demonstrate the cross-packaging of MMTV RNA by MPMV proteins (Figures 2E and 3E).

Similar to the results we obtained for MMTV cross-packaged RNA, MPMV cross-packaged transfer vector (pKAL011 and pSS015) RNAs could not be propagated further as evidenced by the lack of Hyg^r ^colonies or EGFP positive cells in the infected cultures (Table 1). The presence of Hyg^r ^colonies or EGFP positive cells in the infected cultures when MPMV transfer vector (pKAL011 and pSS015) RNAs were packaged by its own proteins (pTR301) assured that the marker genes were efficiently expressed and that there was no compromise on the integrity of our transduction assay. Therefore, it is reasonable to conclude that the absence of Hyg^r ^or EGFP positive cells reflected a true lack of propagation of MPMV cross-packaged vector RNAs. A similar situation has been reported earlier when MPMV RNA could be cross-packaged by heterologous feline immunodeficiency virus (FIV) proteins but these proteins could not further propagate the cross-packaged MPMV RNA [21]. Although not quantitative, the detection levels of the RT-PCR were consistent with the titers obtained in the transduction assay. Using homologous proteins, MPMV transfer vectors (pKAL011 and pSS015) were propagated at much higher efficiency (36,373 CFU/ml and 41% EGFP positive cells) when compared to MMTV transfer vectors (pDA024 and pSS013) (3,676 CFU/ml and 19% EGFP positive cells). Corroborating this observation, MPMV transfer vector RNAs were packaged more efficiently when compared to the same cycle of PCR amplification for MMTV transfer vector RNAs being packaged by its own proteins (Table 1, Figure 2E and 3E).

### Over expressed non-specific RNAs cannot be packaged by MMTV or MPMV proteins

**Figure 4:**
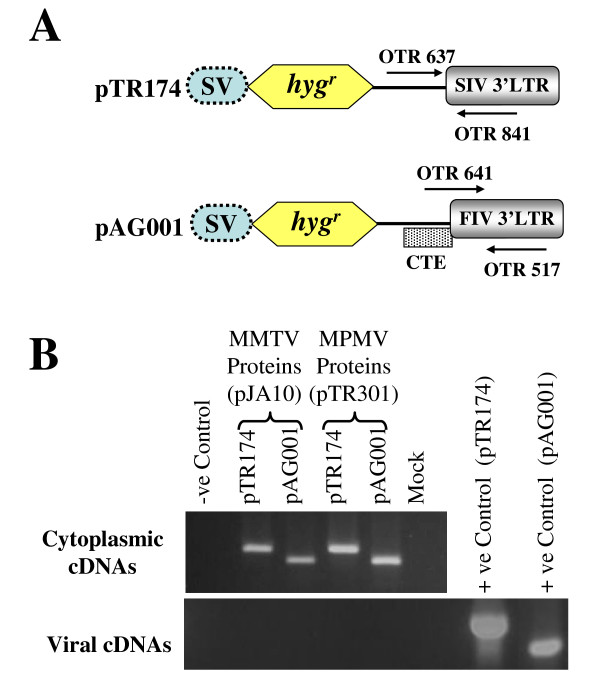
**MMTV and MPMV proteins cannot package non-specific RNAs**. **(A) **Schematic representation of control vectors for non-specific RNA packaging of an over expressed RNA. **(B) **RT-PCR of cytoplasmic (upper panel) and viral (lower panel) cDNA amplified using control vector specific primers. Amplification was conducted for 30 cycles to ensure the amplification of any control vector RNA that may have been non-specifically packaged. While amplifying cytoplasmic and viral cDNAs, primers OTR637 and OTR841 were used for pTR174 and should amplify 368 bp fragment. For pAG001, primers OTR641 and OTR517 were used and should amplify 301 bp fragment.

As an additional control to rule out the possibility of non-specific packaging and possible propagation of random RNAs, we transfected control vectors pTR174 and pAG001 (lacking all the viral sequences at the 5'end) (Figure 4A) separately with MPMV and MMTV packaging constructs (pTR301 and pJA10) along with the envelope expression plasmid MD.G. After taking into consideration all the necessary controls for plasmid DNA contamination, RNA fractionation, and stability of the control transfer vector RNAs (data not shown), we investigated the possibility of packaging and propagation of these non-specific RNAs by both MMTV and MPMV proteins using RT-PCR. Amplification was conducted using control vector specific primers for 30 cycles to ensure the amplification of any control vector RNAs that may have been non-specifically packaged. As shown in figure 4B, neither MPMV nor MMTV proteins could package the control transfer vector RNA (or, if packaged, the level of packaging was below the threshold level of detection), and consequently the vector RNA could not be propagated (Table 1). The lack of packaging of these non-specific RNAs further confirmed our results that MMTV and MPMV proteins show relative specificity in recognizing the heterologous ψ and can efficiently encapsidate the heterologous RNA.

### Comparison between MMTV and MPMV *cis*-acting nucleotide sequences and amino acid sequences important for reverse transcription and integration

**Figure 5:**
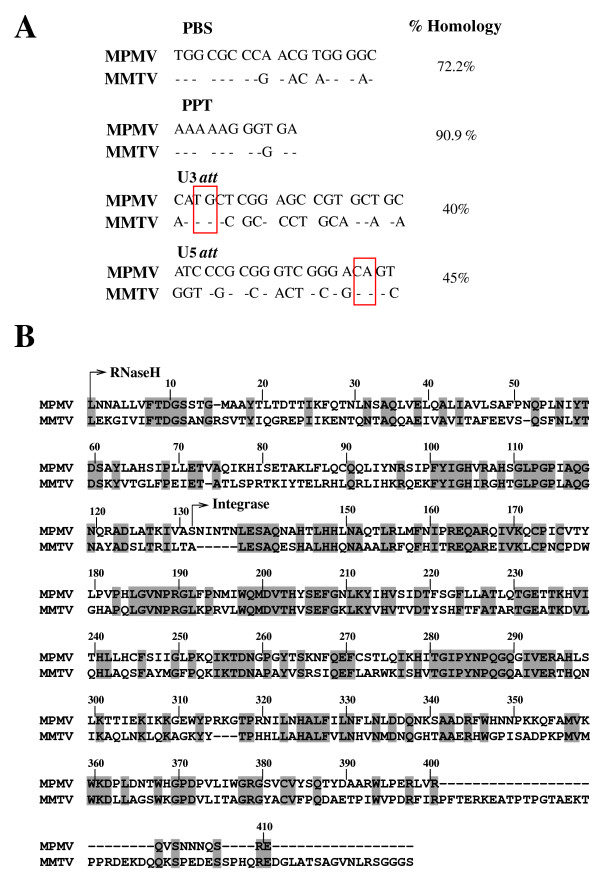
**Nucleotides and amino acid sequences comparison between MMTV and MPMV**. **(A) **Comparison between MMTV and MPMV *cis*-acting sequences needed for successful reverse transcription and integration. PBS, primer binding site; PPT poly purine tract; U3 *att*, attachment site at 3'LTR; U5 *att*, attachment site at 5'LTR. The boxed areas represent the canonical TG and CA dinucleotides in the U3 and U5 *att *sequences. The "-" represents homologous sequences, the differences are represented by the actual nucleotides. **(B) **Amino acid sequence alignment of MPMV RNaseH and integrase with the corresponding region of MMTV using the sequence alignment program, CLUSTAL W. Identical amino acids are boxed.

The reciprocal cross-packaging of MPMV and MMTV RNAs suggests that the ψ of these viruses are readily recognized by each other's NC domain of Gag protein facilitating their efficient cross-encapsidation. However, the absence of Hyg^r ^colonies or EGFP positive cells in the infected cultures suggests a block in post-packaging steps of the viral life cycle such as reverse transcription and/or integration. The successful propagation of MMTV and MPMV transfer vector RNAs would require the recognition of a number of *cis*-acting sequences present on their RNAs by the heterologous proteins following cross-packaging. Of these, reverse transcription and integration would require specific enzymes namely reverse transcriptase (RT) and integrase to work on targets such as PBS/PPT and *att *sites, respectively. Therefore, it is conceivable that MPMV and MMTV enzymes were not able to function on the respective targets of the cross-packaged RNAs resulting in the lack of propagation of these cross-packaged RNAs. With this rationale in mind, we compared the *cis*-acting sequences present on MMTV and MPMV transfer vector RNAs. Our sequence analysis shown in figure 5A revealed that PPT sequences between the two viruses have 90% sequence homology, while PBS showed a sequence homology of approximately 72%. Comparison of *att *sites between the two viruses indicated less than 50% sequence homology (U3 *att *40% whereas the U5 *att *45%).

Regulatory enzymes working on the above-mentioned *cis*-acting sequences may also have played an important role in the inability of the cross-packaged RNAs to propagate because of the sequence heterogeneity between these two viruses. Therefore, RNaseH and integrase were good candidates to be examined carefully since RNaseH plays a pivotal role in hydrolyzing the RNA-DNA hybrid [25] and its inactivation leads to production of non-infectious virus [26]. Similarly, integrase is essential for the integration of the linear retroviral DNA, a crucial step for the completion of the virus life cycle and has also been shown to promote reverse transcription through interactions with the nucleoprotein reverse transcription complex [27]. Thus, using the sequence alignment program, CLUSTAL W [28], we compared the amino acid sequences of MPMV and MMTV RNaseH and integrase. Because of the lack of any published sequences of MMTV integrase and RNaseH, we took the amino acid sequences of MPMV integrase [29] and RNaseH [30] and aligned them with the entire Gag-Pro-Pol polyprotein amino acid sequence of exogenous MMTV(C3H) (accession number AF228552) [31]. As expected, the MPMV RNaseH and integrase sequences aligned with those of the 3'end of Gag-Pro-Pol polyprotein of MMTV revealing varying degrees of heterogeneity. The sequence homology between MPMV RNaseH and integrase with the corresponding sequences of MMTV was found to be 32% and 49%, respectively (Figure 5B).

Based on the sequence analyses of MPMV and MMTV *cis*-acting sequences and those of the regulatory enzymes acting on them (and the fact that both of these viruses utilize Lys tRNA primers; Lys-1, 2 for MPMV and Lys-3 for MMTV), it is plausible to propose that the cross-packaged RNAs have the potential to successfully initiate the process of reverse transcription; however, they may not have been able to efficiently complete this event. In addition to this, the possibility of a fully reverse transcribed unintegrated provirus cannot be ruled out as has been reported earlier [32]. The inability of the cross-packaged RNAs to reverse transcribe and/or to integrate should result in a failed transduction of the target cells because our read out assay is based on the expression of the marker genes from an integrated provirus. This postulation stems from the fact that inadequate integration has been implicated in earlier studies including those involving retroviral cross-packaged RNAs [33,34,21]. Therefore, it is reasonable to propose that due to the great degree of amino acid sequence divergence (in RNaseH and integrase) and the heterogeneity in *att *sequences of these viruses, the cross-packaged RNAs could not be propagated.

### Non-viral RNAs containing MMTV and MPMV packaging sequences can be reciprocally cross-packaged by the heterologous proteins

The results presented so far clearly indicate that MMTV and MPMV proteins were able to recognize the ψ sequence on each other's RNAs. If the recognition of the packaging sequences by the heterologous proteins was sufficient to encapsidate the RNAs, we would argue that any RNA containing these sequences should also be able to be cross-packaged into the homologous as well as the heterologous viral particles. Therefore, the region encompassing sequences containing MPMV [35] and the putative MMTV ψ (unpublished observations) were cloned into expression plasmids that can generate non-viral RNAs, which could act as a substrate for packaging into the assembling virus particles. In a two-plasmid *trans*-complementation assay, the expression plasmids containing the putative MMTV ψ (5'UTR + 400 bp of Gag in the case of pNF007 and R/U5 + 5'UTR + 400 bp of Gag in the case of pNF008; Figure 1A) were transfected along with MMTV (pJA10) as well as MPMV (pTR301) packaging constructs into the 293T producer cells. Similarly, the expression plasmids containing MPMV ψ (R/U5 + 5'UTR + 282 bp of Gag in pND001 and 5'UTR + 282 bp of Gag in pND002; Figure 1B) were also transfected with the homologous MPMV (pTR301) or the heterologous MMTV (pJA10) packaging constructs.

**Figure 6:**
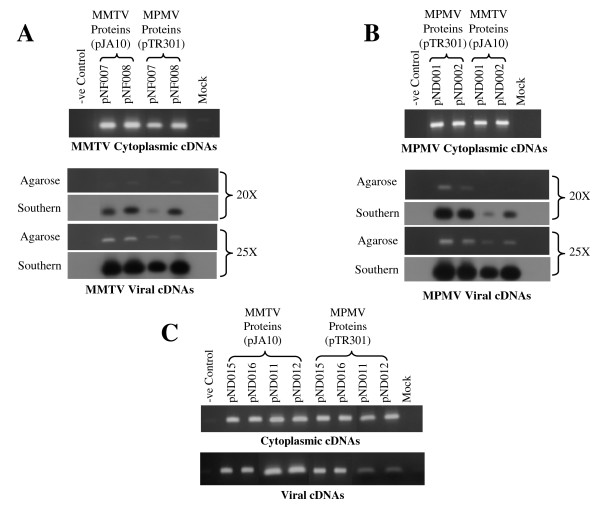
**Heterologous and chimeric transfer vector RNAs cross-packaging**. **(A) **Reciprocal cross-packaging of heterologous RNAs containing MMTV packaging signal. RT-PCR of cytoplasmic (upper panel) and viral (lower panel) cDNAs amplified using MMTV specific primers and probed with the PCR product amplified using the same set of primers and HYB MTV as a template. For this set of experiment, primers OTR567 and OTR560 were used and should amplify 149 bp fragment. **(B) **Reciprocal cross-packaging of heterologous RNAs containing MPMV packaging signals. RT-PCR of cytoplasmic (upper panel) and viral (lower panel) cDNAs amplified using MPMV specific primers and probed with the PCR product amplified using the same set of primers and pKAL011 as template. For this set of experiments, primers OTR112 and OTR197 were used and should amplify 154 bp fragment. **(C) **MMTV and MPMV chimeric transfer vectors RNA containing each other's packaging sequences can be packaged in a reciprocal fashion. Upper panel represents RT-PCR of cytoplasmic cDNAs using vector RNA specific primers to ensure their stable expression. Lower panel represents RT-PCR of viral cDNAs amplified using chimeric vector specific primers. For this set of experiment, primers OTR567 and OTR560 were used for pND011 and pND012 and should amplify 149 bp. For pND015 and pND016, primers OTR730 and OTR197 were used and should amplify 268 bp. However, for the sake of clarity and to follow cross-packaging, the PCR products are shown next to each others in this figure.

Following *trans*-complementation, both cytoplasmic and viral RNAs were isolated and after consideration of the necessary controls (data not shown), the RNAs were reverse transcribed and the RT-PCR was conducted to determine the encapsidation of non-viral RNAs that contain either the MPMV or the putative MMTV ψ by the homologous or the heterologous proteins. RT-PCR analysis conducted on the RNAs demonstrated that non-viral (pNF007 and pNF008) RNAs were packaged by the homologous (MMTV; pJA10) and the heterologous (MPMV; pTR301) proteins (Figure 6A). These results further confirmed our findings of the ability of MPMV proteins to cross-package MMTV transfer vector RNAs in both viral (pDA024 and pSS013) and non-viral (pNF007 and pNF008) context (Figures 2E and 6A). In a reciprocal fashion, MMTV proteins (pJA10) cross-packaged non-viral (pND001 and pND002) RNAs containing MPMV ψ (Figure 6B) confirming our initial observation of the cross-packaging of MPMV transfer vector (pKAL011 and pSS015) RNAs by MMTV proteins in the viral context (Figure 3E). The cross-packaging of non-viral RNA containing ψ further confirmed that the specificity towards RNA packaging was conferred by ψ. Packaging of non-viral RNAs containing the packaging sequences has also been observed for other retroviruses such as bovine leukemia virus (BLV) [36], FIV [24], and MoMLV [37].

### Substitution of the packaging signal in MMTV and MPMV transfer vectors resulted in efficient packaging but drastically reduced vector RNA propagation

The results of the cross-packaging experiments between MMTV and MPMV suggested that the reciprocal cross-packaging was due to the cross-recognition of packaging sequences by the heterologous proteins. The lack of propagation of these cross-packaged transfer vector RNAs, on the other hand, suggested that following packaging some of the events imperative for the transduction of the target cells were not carried out. Therefore, we rationalized that since MPMV proteins are able to recognize the putative MMTV ψ, we should be able to exchange successfully the putative MMTV ψ with that of MPMV in the presence of either MMTV or MPMV PBS (pND015 and pND016 respectively) or vice versa (pND011 and pND012) (Figure 1A and 1B). The chimeric transfer vectors (pND015 and pND016) when co-transfected with MMTV packaging constructs, in addition to being cross-packaged, should also be able to propagate its RNA. Similar strategy has been used to propagate chimeric lentiviral transfer vector RNAs; for example, exchanging the packaging signal in FIV transfer vectors with those of human and simian immunodeficiency virus (HIV and SIV, respectively) resulted not only in packaging but also in the propagation of the chimeric FIV transfer vector RNA by FIV, HIV, and SIV proteins, albeit at a much reduced efficiency [21].

A test of MMTV (pND015 & pND016) and MPMV (pND011 & pND012) chimeric transfer vectors in our *in vivo *packaging and transduction assay using either MMTV (pJA10) or MPMV (pTR301) packaging constructs revealed that the chimeric transfer vector RNAs were successfully packaged and cross-packaged by the respective homologous and heterologous proteins (Figure 6C). However, contrary to our expectations, the packaged RNAs by the homologous or by the heterologous proteins either could not be propagated at all or there was a drastic drop in propagation as evidenced by very few Hyg^r ^colonies (< 25 CFU/ml compared to 3,676 CFU/ml when pDA024 was packaged by pJA10 or 36,373 CFU/ml when pKAL011 was packaged by pTR301; Table 1). Taken together, these rather unexpected results pointed in the direction that we may have disrupted the region around U5/PBS/UTR during the course of exchanging sequences encompassing ψ, and that this disruption proved to be detrimental for vector RNA propagation and not RNA packaging. During the course of cloning these chimeric transfer vectors, an artificial *Not*I site either at PBS/UTR (pND015 and pND011) or at U5/PBS junctions (pND016 and pND012) was created (Figure 1A and 1B). Thus, it is conceivable that such non-natural sequences at these crucial junctions may have affected essential steps in the viral life cycle such as reverse transcription thereby hindering the propagation of these RNAs.

To confirm whether the introduction of such artificial sequences could limit the ability of the transfer vector RNAs to propagate, we created control vectors for both MMTV (pND017 and pND018) and MPMV (pND013 and pND014). In these control transfer vectors, regions encompassing MMTV and MPMV ψ were amplified and cloned back at the same location after artificially creating a *Not*I site as in the case of MMTV (pND015 and pND016) and MPMV (pND011 and pND012) chimeric transfer vectors (Figures 1A and 1B). Test of these control transfer vectors (pND013 and pND017) in which the *Not*I site was created at the PBS/UTR junction revealed that their RNAs were propagated to a much reduced level (20 fold reduction in the case of pND017 when compared to pDA024 and 13 folds reduction in the case of pND013 when compared to pKAL011; Table 1). On the other hand, when *Not*I site was created at the U5/PBS junction (pND014 and pND018), the propagation of these control transfer vectors was further hindered (120 fold reduction in pND018 when compared to pDA024 and 22 fold reduction in pND014 when compared to pKAL011; Table 1). We did not directly investigate the encapsidation of these control transfer vector RNAs, because when similar sequences were exchanged by creating artificial *Not*I site on MMTV and MPMV chimeric transfer vectors (pND015, pND016, pND011, and pND012), their RNAs were shown to be efficiently packaged by both homologous and heterologous proteins (Figure 6C).

The drastic drop in the propagation of the chimeric and the control transfer vectors can be explained on the basis that the introduction of *Not*I site in these vectors may have disrupted RNA structural elements encompassing sequences such as PBS (needed for reverse transcription) and/or sequences encompassing or overlapping ψ (needed for RNA dimerization) and these manipulations in turn may have diminished the chances of viral RNA propagation. Alterations/mutations in dimerization region have been shown to affect multiple steps in the viral life cycle, excluding packaging [38]. For example, in the case of HIV-1, a stem loop structure downstream of the PBS called dimerization initiation site (DIS) is able to dimerize via a "kissing loop" model [[39], and further reviewed in [16]]. In addition, the apical loop of the "kissing loop" hairpin contains a GC-rich autocomplementary sequence called palindrome (pal) [40,41]. Mutations in DIS region and in the palindrome have shown pronounced effects on viral infectivity, minor or no effects on RNA packaging, and variable effects on RNA dimerization [[41,42], and further reviewed in [43]]. Although the existence of a "kissing loop" model and its potential role in the life cycle of MPMV and MMTV has not been established yet, we have observed in close vicinity of the PBS a GC-rich pal sequence in both MPMV (5' UCGCCGGCCGGCGA 3') and MMTV (5' GUCGGCCGAC 3') with 100% autocomplementarity (unpublished observations). In addition, using RNA structure prediction program, we observed putative interaction(s) between *Not*I site (5' GCGGCCGC 3') and PBS (MMTV PBS: 5' UGGCGCCCGAACAGGGA 3'; MPMV PBS: 5' UGGCGCCCAACGUGGGGC 3') or pal in some of the control transfer vectors showing no or much reduced vector RNA propagation (data not shown). The complementarity between these sequences and their GC-rich nature might have disrupted the RNA structural elements in these regions, which compromised the role of these biologically important sequences. Furthermore, It has earlier been reported that the interaction between the PBS and the tRNA 3' terminus is crucial for primer selection in MLV reverse transcription and that mutations in PBS of both MLV and HIV-1 involving aberrant reverse transcription have been reported to hinder viral replication [44]; consequently, the interaction between the *Not*I site and PBS may have hindered the accessibility of tRNA to PBS in our control and chimeric transfer vectors. Therefore it is reasonable to propose that the substitution and/or introduction of the heterologous sequences, in addition to the creation of the *Not*I site, may have disrupted the yet to be identified "kissing loop" model in the case of MPMV and MMTV, which thwarted post RNA packaging steps of retroviral life cycle, resulting in the abrogation and/or much reduced propagation of the packaged RNAs in the control as well as chimeric transfer vectors.

Owing to the wide use of retroviral vectors in human gene therapy and the safety issues related with cross- and co-packaging among retroviruses, these areas have been extensively investigated and have revealed that reciprocal as well as non-reciprocal cross-packaging among genetically distinct, simple and complex, retroviruses can take place [17,21,45-54]. Not only cross-packaging but also co-packaging, resulting in the exchange of genetic information, has been reported in genetically distant retroviruses such as SNV and MLV [17] and HIV-1 and HIV-2 [18]. One of the most important consequences of exchanging genetic information is the generation of viral variants with unknown pathogenic potential. This has brought the vector safety concerns to the forefront especially in the light of the mobilization of HIV-1 based vectors from the transduced cells following infection with the wild type virus [55,56]. In addition, the generation of a replication competent retrovirus through the process of recombination between the vector, the packaging construct, and endogenous retrovirus-like elements has also been reported [57]. In light of these safety concerns, we tested whether another non-primate retrovirus candidate for human gene therapy, MMTV, would be prone to cross-packaging with a primate retrovirus as has previously been shown that a phylogenetically distant non-primate lentivirus (FIV) could be packaged by primate lentiviruses [21]. Our study showed that MMTV and MPMV packaging sequences are promiscuous and could be recognized by each other's proteins suggesting potential RNA-protein interactions among divergent retroviruses. It would be interesting to determine how frequently MMTV RNA can be cross-packaged by other retroviral proteins and whether there are obstacles to these RNA-protein interactions. These observations raise the possibility that MMTV and MPMV RNAs could also co-package and exchange genetic information. Therefore, it would be interesting to investigate whether co-packaging between MMTV and MPMV could occur and if so, at what frequency and what are the properties of the resulting recombinant variants, if at all.

## Conclusion

The results presented in this study raise important questions about the design and use of candidate vectors for human gene therapy and suggest that sequences, which could contribute to such cross-packaging, should be eliminated from the transfer vectors to allow the establishment of safer and more effective vectors for human gene transfer studies. The retrovirus life cycle may be exploited to render the *cis*-acting sequences of an integrated transfer vector inactive through a process called self-inactivation (SIN) [45,55,58-61]. In addition to SIN vectors, a novel super-split packaging system has also been recently developed, which incorporates many new safety features. In such a system, *gag *was separated from *pol *and the protease was provided in *trans *by a less cytotoxic mutant, while maintaining high titers [62]. Surprisingly, despite taking into consideration several safety aspects in modified packaging systems and transfer vectors, vector mobilization as well as leaky transcriptional termination have been reported [63-65]. Therefore, in order to construct efficient and safe vectors, a thorough understanding of RNA packaging and cross packaging among retroviruses is imperative, and the understanding we will gain from such studies should allow employing multiple strategies to prevent viral replication through packaging and/or recombination after delivery of the gene of interest into the target cells.

## Methods

### Numbering system

Nucleotide designation for MMTV is based on HYB MTV molecular clone sequence [66]. Nucleotide designation for MPMV is based on GenBank accession number M12349[67].

### Plasmid Constructions

#### MMTV packaging constructs and transfer vectors

The MMTV packaging construct, pJA10, has been described previously [20]. Briefly, pJA10 expresses MMTV *gag/pol *gene from hCMV promoter and contains constitutive transport element (CTE) from MPMV downstream of MMTV *gag/pol*. pDA024 and pSS013 are MMTV based transfer vectors that have been described previously [20] and contain 5' chimeric LTR in which MMTV promoter sequences have been replaced with that of hCMV (Figure 1A).

To asses whether the presence of regions encompassing the putative MMTV ψ on non-viral RNAs would facilitate the packaging of these RNAs, sequences containing the putative MMTV ψ (5'UTR and 400 bp of Gag) and (R/U5/5'UTR and 400 bp of Gag) were amplified using sense primers OTR680 and OTR617 and an anti-sense primer OTR552 using HYB MTV as a template. The PCR products were cleaved with the artificially created *Hind*III and *Spe*I sites and cloned into *Hind*III and *Xba*I sites of pNF003 (a derivative of pcDNA3) generating pNF007 and pNF008, respectively (Figure 1A).

pND015 and pND016 are pDA024 derivatives containing MPMV ψ either in the presence (pND015) or absence (pND016) of MMTV PBS and were generated through series of cloning steps. Briefly, the 3'end of MMTV genome containing SV-*hyg*^*r *^cassette and MPMV CTE along with 3'LTR was obtained by digesting pDA024 with *Spe*I and *Kpn*I. The 5'region of MMTV genome containing 5'LTR with and without PBS was amplified using sense primer OTR617 and anti-sense primers OTR727 and OTR728 and pDA024 as a template. The resulting PCR products were digested with the artificially created *Sac*I and *Spe*I sites. The resulting *Sac*I-*Spe*I PCR fragment containing 5'end of the genome (without the packaging signal) and *Spe*I-*Kpn*I fragment containing the 3'end of the genome were ligated in a three way ligation to a pIC based cloning vector [68], which had already been cleaved with *Sac*I and *Kpn*I resulting in pND005 and pND006, respectively. Such a cloning strategy created an artificial *Not*I site at the junction of PBS/UTR in the case of pND005 and at the U5/PBS junction in the case of pND006. Next, sequences encompassing MPMV packaging determinants (UTR and 282 bp of Gag) with and without MPMV PBS were amplified using sense primer OTR730 and OTR729 and OTR731 as anti-sense primers. The resulting PCR products were cleaved by the flanking artificially created *Not*I sites and were cloned into the *Not*I site of pND005 and pND006 resulting in the final clones pND015 and pND016, respectively (Figure 1A).

To ensure that the creation of an artificial *Not*I site in the region encompassing MMTV packaging sequences will not affect RNA packaging or propagation adversely, we created pND017 and pND018 as control transfer vectors. Putative sequences that are involved in MMTV RNA packaging with and without the MMTV PBS were amplified using sense primers OTR724 and OTR725 and anti-sense primer OTR726 and pDA024 as a template. The combination of these primers created flanking artificial *Not*I sites in the PCR products facilitating their cloning into *Not*I site of pND005 and pND006 to generate pND017 and pND018, respectively (Figure 1A).

#### MPMV packaging constructs and transfer vectors

The MPMV packaging construct, pTR301, has already been described [21]. Briefly, it expresses MPMV *gag/pol *genes from hCMV intron A promoter/enhancer and contains MPMV CTE between Pol termination codon and bovine growth hormone (BGH) Poly (A) sequences. The MPMV transfer vector, pKAL011, has also been described previously [21] and contains all *cis*-acting sequences necessary for RNA packaging and propagation. pSS015 is similar to pKAL011 except it contains SV-*EGFP *cassette instead of SV-*hyg*^*r *^cassette (Figure 1B).

To determine if the presence of regions encompassing MPMV packaging sequences on non-viral RNAs would facilitate the packaging of these RNAs, two different regions encompassing MPMV ψ were cloned into pNF003. A region encompassing MPMV ψ (R/U5, 5'UTR, and 282 bp of Gag) was amplified using a sense primer OTR615, an anti-sense primer OTR616, and pKAL011 as a template. The PCR product was cleaved with the artificially created *Hind*III and *Nhe*I sites and was cloned into *Hind*III and *Xba*I sites of pNF003 generating pND001. pND002 is another version of pcDNA3 based clone containing MPMV ψ and was created by isolating *Sfo*I-*Hpa*I fragment containing MPMV PBS, 5'UTR and 282 bp of Gag from pKAL011 and was cloned into the blunted *Hind*III and *Xba*I sites of NF003 (Figure 1B).

pND011 and pND012 are MPMV based vectors in which MPMV ψ has been replaced with that of MMTV either in the presence (pND011) or absence (pND012) of MPMV PBS and were created through series of cloning steps. As a first step, the 3'end of MPMV genome containing 3'LTR was obtained by digesting pKAL011 with *Nhe*I and *BamH*I. The MPMV 5'LTR along with or without PBS were amplified using sense primer OTR721 and anti-sense primers OTR722 and OTR723 using pKAL011 as a template. The resulting *Xho*I-*Nhe*I PCR fragments containing 5'end of the genome (without the packaging signal) and *Nhe*I and *BamH*I fragment containing the 3'end of the genome were ligated in a three way ligation at the *Xho*I and *BamH*I sites of a pIC based cloning vector resulting in pND003 and pND004, respectively. Similar to the cloning scheme of MMTV chimeric vectors, such a cloning strategy created an artificial *Not*I site at the PBS/UTR and U5/PBS junctions in the case of pND003 and pND004, respectively. Next, the putative MMTV ψ (5'UTR and 400 bp of Gag) with and without MMTV PBS were amplified from pDA024 using sense primers OTR725 and OTR724 and OTR726 as the anti-sense primer. The resulting PCR products were cleaved by the flanking artificially created *Not*I sites and were cloned into the *Not*I site of pND003 and pND004 resulting in pND007 and pND008. Finally, SV-*hyg*^*r *^cassette containing flanking *Nhe*I sites was cloned into the *Nhe*I site of pND007 and pND008 generating pND011 and pND012 (Figure 1B).

pND013 and pND014 were created as controls to see the effects of an artificially created *Not*I site within MPMV ψ. Regions containing MPMV ψ (with and without the PBS) were amplified using sense primers OTR729 and OTR730 and anti-sense primer OTR731 and pKAL011 as a template. The PCR products were digested with the flanking *Not*I site and cloned into the *Not*I site of pND003 and pND004, respectively generating pND009 and pND010. Finally, SV-*hyg*^*r *^cassette containing flanking *Nhe*I sites was cloned into *Nhe*I site of pND009 and pND010 generating the final clones pND013 and pND014 (Figure 1B).

#### Plasmids for nonspecific RNA packaging

pTR174 has been described previously [49] and was created to test the possibility of non-specific packaging of an over expressed RNA containing the SV-*hyg*^*r *^cassette through "retrofection" [69,70] and uses SIV 3'LTR for transcript termination. pAG001 is similar to pTR174 except that at the 3'end it contains FIV 3'LTR for transcript termination. Both of these control vectors lack all of the viral sequences at the 5'end (Figure 4A).

#### Envelope expression construct

All these transfer vectors were psuedotyped in a three plasmid *trans*-complementation assay using vesicular stomatitis virus envelope G (VSV-G) expression plasmid (MD.G) that has been previously described [22].

### Transfection and infection of cells

The producer 293T cells were maintained and transfected by the calcium phosphate method as described earlier [71]. Seventy two hours post-transfection, viral supernatants were harvested for pelleting virus particles to isolate viral RNA and some portion was used to infect HeLa CD4^+ ^cells as described previously [21]. Forty-eight hours following infection, cells that were infected with transfer vectors containing *hygromycin resistance *gene were selected with media containing hygromycin B phosphotranferase. Hyg^r ^colonies that appeared were stained and counted after 9–11 days as described previously [21]. Target cells that were infected with transfer vectors containing *EGFP *gene were trypsinized 48 hours post-infection, washed with PBS, and analyzed on a Becton Dickinson Fluorescent Activating Cell Sorting (FACS) using the CellQuest software.

### Ultracentrifugation of virus particles

Viral supernatants collected seventy-two hours post-transfection were subjected to a low-speed centrifugation (4000 rpm for 10 minutes) for removal of cellular debris. Following which, viral particles were filtered using 0.2-micron syringe filters and ultracentrifuged using 20% sucrose cushion (using SW41 rotor at 26,000 rpm for 2 hours at 4°C) as described previously [24].

### RNA isolation and Reverse Transcriptase Polymerase Chain Reaction (RT-PCR)

The transfected cells were taken off from the culture plates without trypsinization and fractionated into cytoplasmic and nuclear RNA fractions as described earlier [71]. For viral RNA isolation, pelleted virions resuspended in TNE buffer were lysed in 500 μl of Trizol LS reagent containing 8 μl of polyacryl (used as a carrier) as described earlier [24]. Following RNA isolation, both cytoplasmic and viral RNAs were DNase treated and tested by PCR to confirm the absence of any contaminating plasmid DNA that may have been carried over from the transfected cultures. After confirming that RNA preparations were devoid of any contaminating DNA, cytoplasmic and viral RNAs were reverse transcribed and amplified. cDNA prepared from the cytoplasmic RNA fractions were amplified using vector specific primers to monitor the expression of the transfer vector RNA in the cells. In addition, cytoplasmic fractions were also amplified to confirm the integrity of the fractionation process in a multiplex PCR in the presence of primers/competimers for 18S ribosomal RNA as a control for the presence of amplifiable cDNA in the PCR reactions (18S Quantum competimer control, Ambion, Austin, TX). Viral cDNAs were amplified using vector specific primer to determine the packaging of the transfer vector RNA. Amplified PCR products were analyzed on 2–4% agarose gels and, in some cases, the gels were further processed for Southern blot analysis as described previously [24]. Details of the primers used in cloning, RT-PCR, and Southern hybridization can be obtained from authors upon request.

## Competing interests

The authors declare that they have no competing interests.

## Authors' contributions

NSD, PSP, AG, JA, EB, and TAR performed the experiments (cloning, *trans *complementation assay, RNA extraction, RT-PCR, Southern blotting). SAJ performed RNase H and Integrase amino acid sequences alignment. NSD and TAR conceived the study and participated in its design. All authors read and approved the final manuscript.

## References

[B1] Mustafa F, Lozaon M, Dudley JP (2000). C3H mouse mammary tumor virus superantigen function requires a splice donor in the envelope gene. J Virol.

[B2] Mustafa F, Bhadra S, Johnston D, Lozano M, Dudley JP (2003). The type B leukemogenic virus superantigen is dispensable for T-cell lymphomagenesis. J Virol.

[B3] Acha-Orbea H, Palmer E (1991). Mls-a retrovirus exploits the immune system. Immunol Today.

[B4] Marrack P, Kushnir E, Kappler J (1991). A maternally inherited superantigen encoded by a mammary tumour virus. Nature.

[B5] Salmons B, Erfle V, Brem G, Günzburg WH (1990). Naf, a trans-regulating negative-cis acting factor encoded within the mouse mammary tumor virus open reading frame region. J Virol.

[B6] Indik S, Günzburg WH, Salmons B, Rouault F (2005). Mouse mammary tumor virus infects human cells. Cancer Res.

[B7] Mertz JA, Simper MS, Lozano MM, Payne SM, Dudley JP (2005). Mouse mammary tumor virus encodes a self-regulatory RNA export protein and is a complex retrovirus. J Virol.

[B8] Müllner M, Salmons B, Günzburg WH, Indik S (2008). Identification of the Rem-responsive element of mouse mammary tumor virus. Nucleic Acids Res.

[B9] Mertz JA, Lozano MM, Dudley JP (2009). Rev and Rex proteins of human complex retroviruses function with the MMTV Rem-responsive element. Retrovirology.

[B10] Arroyo J, Winchester E, McLellan BS, Huber BT (1997). Shared promoter elements between a viral superantigen and the major histocompatibility complex class II-associated invariant chain. J Virol.

[B11] Günzburg WH, Heinemann F, Wintersperger S, Miethke T, Wagner H, Erfle V, Salmons B (1993). Endogenous superantigen expression controlled by a novel promoter in the MMTV long terminal repeat. Nature.

[B12] Miller CL, Garner R, Paetkau V (1992). An activation-dependent, T-lymphocyte-specific transcriptional activator in the mouse mammary tumor virus env gene. Mol Cell Biol.

[B13] Ham J, Thomson A, Needham M, Webb P, Parker M (1988). Characterization of response elements for androgens, glucocorticoids and progestins in mouse mammary tumour virus. Nucleic Acids Res.

[B14] Klein R, Ruttkowski B, Schwab S, Peterbauer T, Salmons B, Günzburg WH, Hohenadl C (2008). Mouse mammary tumor virus promoter-containing retroviral promoter conversion vectors for gene-directed enzyme prodrug therapy are functional in vitro and in vivo. J Biomed Biotechnol.

[B15] D'Souza V, Summers MF (2005). How retroviruses select their genomes. Nat Rev Microbiol.

[B16] Lever AML (2007). HIV RNA packaging. Adv Pharmacol.

[B17] Yin PH, Hu W-S (1997). RNAs from genetically distinct retroviruses can copackage and exchange genetic information in vivo. J Virol.

[B18] Motomura K, Chen J, Hu WS (2008). Genetic recombination between human immunodeficiency virus type 1 (HIV-1) and HIV-2, two distinct human lentiviruses. J Virol.

[B19] Günzburg WH, Salmons B (1986). Mouse mammary tumor virus mediated transfer and expression of neomycin resistance to infected cultured cells. Virology.

[B20] Rizvi TA, Ali J, Phillip PS, Ghazawi A, Mustafa F (2009). Role of a heterologous retroviral transport element in the development of genetic complementation assay for mouse mammary tumor virus (MMTV) replication. Virology.

[B21] Browning MT, Schmidt RD, Lew KA, Rizvi TA (2001). Primate and feline lentivirus vector RNA packaging and propagation by heterologous lentivirus virions. J Virol.

[B22] Naldini L, Blomer U, Gallay P, Ory D, Mulligan R, Gage FH, Verma IM, Trono D (1996). *In vivo *gene delivery and stable transduction of nondividing cells by a lentiviral vector. Science.

[B23] Tan W, Felber BK, Zolotukhin AS, Pavlakis GN, Schwartz S (1995). Efficient expression of the human papillomavirus type 16 L1 protein in epithelial cells by using Rev and the Rev-responsive element of human immunodeficiency virus or the cis-acting transactivation element of simian retrovirus type 1. J Virol.

[B24] Ghazawi A, Mustafa F, Phillip PS, Jayanth P, Ali J, Rizvi TA (2006). Both the 5' and 3' LTRs of FIV contain minor RNA encapsidation determinants compared to the two core packaging determinants within the 5' untranslated region and gag. Microbes Infect.

[B25] Schatz O, Mous J, Le Grice SF (1990). HIV-1 RT-associated ribonuclease H displays both endonuclease and 3'-5' exonuclease activity. EMBO J.

[B26] Garcés J, Wittek R (1991). Reverse-transcriptase-associated RNaseH activity mediates template switching during reverse transcription in vitro. Proc Biol Sci.

[B27] Wu X, Liu H, Xiao H, Conway JA, Hehl E, Kalpana GV, Prasad V, Kappes JC (1999). Human immunodeficiency virus type 1 integrase protein promotes reverse transcription through specific interactions with the nucleoprotein reverse transcription complex. J Virol.

[B28] Thompson JD, Higgins DG, Gibson TJ (1994). CLUSTAL W: improving the sensitivity of progressive multiple sequence alignment through sequence weighting, position-specific gap penalties and weight matrix choice. Nucleic Acids Res.

[B29] Snásel J, Krejcík Z, Jencová V, Rosenberg I, Ruml T, Alexandratos J, Gustchina A, Pichová I (2005). Integrase of Mason-Pfizer monkey virus. FEBS J.

[B30] Kanaya S, Kohara A, Miura Y, Sekiguchi A, Iwai S, Inoue H, Ohtsuka E, Ikehara M (1990). Identification of the amino acid residues involved in an active site of Escherichia coli ribonuclease H by site-directed mutagenesis. J Biol Chem.

[B31] Hook LM, Agafonova Y, Ross SR, Turner SJ, Golovkina TV (2000). Genetic of mouse mammary tumor virus-induced mammary tumors: linkage of tumor induction to the gag gene. J Virol.

[B32] Lewis PF, Emerman M (1994). Passage through mitosis is required for oncoretroviruses but not for the human immunodeficiency virus. J Virol.

[B33] Masuda T, Kuroda MJ, Harada S (1998). Specific and independent recognition of U3 and U5 *att *sites by human immunodeficiency virus type 1 integrase in vivo. J Virol.

[B34] Hlavaty J, Stracke A, Klein D, Salmons B, Günzburg WH, Renner M (2004). Multiple modifications allow high-titer production of retroviral vectors carrying heterologous regulatory elements. J Virol.

[B35] Schmidt RD, Mustafa F, Lew KA, Browning MT, Rizvi TA (2003). Sequences within both the 5' untranslated region and the gag gene are important for efficient encapsidation of Mason-Pfizer monkey virus RNA. Virology.

[B36] Jewell NA, Mansky LM (2005). Packaging of heterologous RNAs by a minimal bovine leukemia virus RNA packaging signal into virus particles. Arch Virol.

[B37] Adam MA, Miller AD (1988). Identification of a signal in a murine retrovirus that is sufficient for packaging of nonretroviral RNA into virions. J Virol.

[B38] Paillart JC, Berthoux L, Ottmann M, Darlix JL, Marquet R, Ehresmann B, Ehresmann C (1996). A dual role of the putative RNA dimerization initiation site of human immunodeficiency virus type 1 in genomic RNA packaging and proviral DNA synthesis. J Virol.

[B39] Paillart JC, Skripkin E, Ehresmann B, Ehresmann C, Marquet R (1996). A loop-loop "kissing" complex is the essential part of the dimer linkage of genomic HIV-1 RNA. Proc Natl Acad Sci USA.

[B40] Clever JL, Wong ML, Parslow TG (1996). Requirements for kissing-loop-mediated dimerization of human immunodeficiency virus RNA. J Virol.

[B41] Shen N, Jetté L, Liang C, Wainberg MA, Laughrea M (2000). Impact of human immunodeficiency virus type 1 RNA dimerization on viral infectivity and of stem-loop B on RNA dimerization and reverse transcription and dissociation of dimerization from packaging. J Virol.

[B42] Laughrea M, Shen N, Jetté L, Wainberg MA (1999). Variant effects of non-native kissing-loop hairpin palindromes on HIV replication and HIV RNA dimerization: role of stem-loop B in HIV replication and HIV RNA dimerization. Biochemistry.

[B43] Swanstorm R, Wills JW, Coffin JM, Hughes SH, Varmus HE (1997). Synthesis, assembly, and processing of viral proteins. Retroviruses.

[B44] Mikkelsen JG, Lund AH, Kristensen KD, Duch M, Sørensen MS, Jørgensen P, Pedersen FS (1996). A preferred region for recombinational patch repair in the 5' untranslated region of primer binding site-impaired murine leukemia virus vectors. J Virol.

[B45] Dougherty JP, Wisniewski R, Yang SL, Rhode BW, Temin HM (1989). New retrovirus helper cells with almost no nucleotide sequence homology to retrovirus vectors. J Virol.

[B46] Embertson JE, Temin HM (1987). Lack of competition results in efficient packaging of heterologous murine retroviral RNAs and reticuloendotheliosis virus encapsidation-minus RNAs by the reticuloendotheliosis virus helper cell line. J Virol.

[B47] Yang S, Temin HM (1994). A double hairpin structure is necessary for the efficient encapsidation of spleen necrosis virus retroviral RNA. EMBO J.

[B48] Kewalramani VN, Panganiban AT, Emerman M (1992). Spleen necrosis virus, an avian immunosuppressive retrovirus, shares a receptor with the type D simian retroviruses. J Virol.

[B49] Rizvi TA, Panganiban A (1993). Simian immunodeficiency virus RNA is efficiently encapsidated by human immunodeficiency virus type 1 particles. J Virol.

[B50] White SM, Renda M, Nam N-Y, Klimatcheva E, Zhu Y, Fisk J, Halterman M, Rimel B, Federoff H, Pandya S, Rosenblatt JD, Planelles V (1999). Lentivirus vectors using human and simian immunodeficiency virus elements. J Virol.

[B51] Kaye JF, Lever AM (1998). Nonreciprocal packaging of human immunodeficiency virus type 1 and type 2 RNA: a possible role for the p2 domain of gag in RNA encapsidation. J Virol.

[B52] Strappe PM, Hampton DW, Brown D, Cachon-Gonzalez B, Caldwell M, Fawcett JW, Lever AM (2005). Identification of unique reciprocal and non reciprocal cross packaging relationships between HIV-1, HIV-2 and SIV reveals an efficient SIV/HIV-2 lentiviral vector system with highly favourable features for in vivo testing and clinical usage. Retrovirology.

[B53] Certo JL, Shook BF, Yin PD, Snider JT, Hu W-S (1998). Nonreciprocal pseudotyping: murine leukemia virus proteins cannot efficiently package spleen necrosis virus-based vector RNA. J Virol.

[B54] Parveen Z, Mukhtar M, Goodrich A, Acheampong E, Dornburg R, Pomerantz RJ (2004). Cross-packaging of human immunodeficiency virus type 1 vector RNA by spleen necrosis virus proteins: construction of a new generation of spleen necrosis virus-derived retroviral vectors. J Virol.

[B55] Bukovsky AA, Song J, Naldini L (1999). Interaction of human immunodeficiency virus-derived vectors with wild-type virus in transduced cells. J Virol.

[B56] Evans JT, Garcia JV (2000). Lentivirus vector mobilization and spread by human immunodeficiency virus. Hum Gene Ther.

[B57] Chong H, Starkey W, Vile RG (1998). A replication-competent retrovirus arising from a split-function packaging cell line was generated by recombination events between the vector, one of the packaging constructs, and endogenous retroviral sequences. J Virol.

[B58] Delviks KA, Hu W-S, Pathak VK (1997). Ψ^- ^vectors: murine leukemia virus-based self-inactivating and self-activating retroviral vectors. J Virol.

[B59] Dull T, Zufferey R, Kelly M, Mandel RJ, Nguyen M, Trono D, Naldini L (1998). A third generation of lentivirus vector with a conditional packaging system. J Virol.

[B60] Ismail SI, Kingsman SM, Kingsman AJ, Uden M (2000). Split-intron retroviral vectors: Enhanced expression with improved safety. J Virol.

[B61] Zufferey R, Dull T, Mandel RJ, Bukovsky A, Quiroz D, Naldini L, Trono D (1998). Self-inactivating lentivirus vectors for safe and efficient *in vivo *gene delivery. J Virol.

[B62] Westerman KA, Zhujun Ao, Cohen EA, Leboulch P (2007). Design of a trans protease lentiviral packaging system that produces high titer virus. Retrovirology.

[B63] Logan AC, Haas DL, Kafri T, Kohn DB (2004). Integrated self-inactivating lentiviral vectors produce full-length genomic transcripts competent for encapsidation and integration. J Virol.

[B64] Hanawa H, Persons DA, Nienhuis AW (2005). Mobilization and mechanism of transcription of integrated self-inactivating lentiviral vectors. J Virol.

[B65] Schambach A, Galla M, Maetzig T, Loew R, Baum C (2007). Improving transcriptional termination of self-inactivating gamma-retroviral and lentiviral vectors. Mol Ther.

[B66] Shackleford GM, Varmus HE (1988). Construction of a clonable, infectious, and tumorigenic mouse mammary tumor virus provirus and a derivative genetic vector. Proc Natl Acad Sci USA.

[B67] Sonigo P, Barker CS, Hunter E, Hobson S-W (1986). Nucleotide sequences of Mason-Pfizer monkey virus: An immunosuppressive D-type retrovirus. Cell.

[B68] Marsh JL, Erfle M, Wykes EJ (1984). The pIC plasmid and phage vectors with versatile cloning sites for recombinant selection by insertional inactivation. Gene.

[B69] Dornburg R, Temin HM (1988). Retroviral vector system for the study of cDNA gene formation. Mol Cell Biol.

[B70] Linial M (1987). Creation of a processed pseudogene by retroviral infection. Cell.

[B71] Mustafa F, Jayanth P, Phillip PS, Ghazawi A, Schmidt RD, Lew KA, Rizvi TA (2005). Relative activity of the feline immunodeficiency virus promoter in feline and primate cell lines. Microbes Infect.

